# MG‐PE3 bioinspired nanoparticle treatment for clearing amyloid with no toxicity

**DOI:** 10.1002/alz.088444

**Published:** 2025-01-09

**Authors:** Hyuck Kim, Jeong‐Kee Yoon, Wooseung Lee, Miyeon Jeon, Hyunju Lee, Somang Kang, Ee‐Seul Kang, Ji Eun Lee, Seyeon Kim, Hoon Suk Rho, Allan I. Levey, YongTae Kim

**Affiliations:** ^1^ MEPSGEN, Seoul Korea, Republic of (South); ^2^ Emory University, Atlanta, GA USA; ^3^ Emory University School of Medicine, Atlanta, GA USA

## Abstract

**Background:**

Impaired Aβ clearance plays a key role in the common, late‐onset AD. Anti‐Aβ immunotherapies are controversial, in part because of high rates of serious side effects including edema, microhemorrhages, and siderosis, highlighting the importance of the development of alternative Aβ clearance strategy. Here, we introduce a bioinspired nanoparticle named MG‐PE3 crossing the human blood‐brain barrier (BBB) and clearing Aβ with no adverse effect. We used a human BBB chip to test the BBB‐crossing capability in vitro and AD mouse model (5xFAD) to evaluate the Aβ clearance efficacy and the potential toxicity. The good BBB penetration, enhanced glial uptake, and high Aβ affinity of MG‐PE3 resulted in effective clearance of Aβ with no toxicity, thus providing an excellent potential to overcome the current challenges for AD treatment.

**Method:**

Microfluidic technology was used to produce MG‐PE3 with uniform physicochemical properties. MG‐PE3 was tested in vitro for its binding affinity to Aβ, clearance ability with Aβ aggregates, and degradation by glia. MG‐PE3 (vs. saline) was then tested in vivo for Aβ clearance performance, in which MG‐PE3 was intravenously injected to 3‐month‐old wild type (WT) and 5xFAD mice at every third day for 3 months. The BBB penetration was examined by IVIS and the chronic efficacy on AD pathology was assessed by analyzing Aβ levels in CSF and plasma, Aβ load, and neuroinflammation.

**Result:**

Aβ aggregation was significantly inhibited in vitro by MG‐PE3. Further, IVIS imaging presented that MG‐PE3 was delivered into the brain of WT and 5xFAD in a concentration‐dependent manner and superior BBB permeability of MG‐PE3 than apoE3. Compared to saline‐treated groups, MG‐PE3‐treated 5xFAD groups showed significantly lower levels of Aβ40 and Aβ42 in plasma and CSF and, more importantly, reduced Aβ burden in cortex and hippocampus, leading to decreased expression of cytokines. Moreover, no evidence of abnormality and toxic effects were observed in major organs in MG‐PE3‐treated 5xFAD.

**Conclusion:**

MG‐PE3, a bioinspired nanoparticle manufacturable with good homogeneity, showed enhanced BBB penetration, effective brain Aβ clearance, and low risk of adverse effects, with excellent potential for ameliorating amyloidosis and AD pathogenesis. Overall, MG‐PE3 is the first therapeutic nanomedicine for promising disease‐modifying treatment for AD.

